# Bright-field to fluorescence microscopy image translation for cell nuclei health quantification

**DOI:** 10.1017/S2633903X23000120

**Published:** 2023-06-15

**Authors:** Ruixiong Wang, Daniel Butt, Stephen Cross, Paul Verkade, Alin Achim

**Affiliations:** 1 Visual Information Laboratory, University of Bristol, Bristol, United Kingdom; 2 School of Biochemistry, University of Bristol, Bristol, United Kingdom; 3 Wolfson Bioimaging Facility, University of Bristol, Bristol, United Kingdom

**Keywords:** attention network, fluorescent image translation, GAN, nuclei classification

## Abstract

Microscopy is a widely used method in biological research to observe the morphology and structure of cells. Amongst the plethora of microscopy techniques, fluorescent labeling with dyes or antibodies is the most popular method for revealing specific cellular organelles. However, fluorescent labeling also introduces new challenges to cellular observation, as it increases the workload, and the process may result in nonspecific labeling. Recent advances in deep visual learning have shown that there are systematic relationships between fluorescent and bright-field images, thus facilitating image translation between the two. In this article, we propose the cross-attention conditional generative adversarial network (XAcGAN) model. It employs state-of-the-art GANs (GANs) to solve the image translation task. The model uses supervised learning and combines attention-based networks to explore spatial information during translation. In addition, we demonstrate the successful application of XAcGAN to infer the health state of translated nuclei from bright-field microscopy images. The results show that our approach achieves excellent performance both in terms of image translation and nuclei state inference.

## Impact Statement

Fluorescent images reveal information that cannot be revealed from bright-field images. Fluorescent image acquisition is a well-established technique but also complex. It requires sample preparation and can be time-consuming and error-prone. We present an image translation method based on deep learning which allows for revealing concealed information in bright-field images. The method has a high working efficiency, and whose speed of translating a fluorescent image ( 
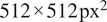
 ) from a bright-field microscopy image stack can be less than a minute using publicly available Python libraries (mainly PyTorch) on mid-range, consumer hardware. In addition, our model is also able to infer the “health” state of nuclei from bright-field microscopy images simultaneous to the image translation process. Our method presents an alternative method for fluorescent image acquisition in biological research, for instance, where fluorescent labeling is not possible.

## 1. Introduction

Microscopy is a fundamental method for visualizing and interpreting cellular structures and processes invisible to the eye. Since around 1280 AD, the field of optics has evolved to go beyond the limits of human vision, eventually leading to the creation of simple single-lens microscopes^(^^1^ ^)^. Scientistcs’ understanding of light, optics, and materials has improved throughout the centuries and have brought forth far more powerful imaging technologies such as confocal laser scanning, fluorescent, electron, X-ray, and even acoustic microscopy^(^^1^ ^)^. Some cellular micro-structures are concealed in transmitted-light images, such as those acquired via bright-field, differential interference contrast (DIC) and phase contrast (PC) imaging. Fluorescent labeling can compensate for this limitation by highlighting specific cellular structures and can allow for their real-time tracking. The principle of fluorescence can be summarized as follows: electrons absorb energy from a photon (such as provided by the light source/laser beam), electrons become excited and rise to a higher energy level, and finally, the electrons lose their energy (as visible light) and return to their original energy level^(^^2^ ^)^. Materials that display fluorescent properties are classified as fluorophores. Fluorophores can consist of fluorescent chemicals (e.g., nuclear dyes such as DAPI and Hoechst) and fluorescent proteins (e.g., green fluorescent protein [GFP]) bound to antibodies^(^^3^ ^,^^4^ ^)^. Additionally, some endogenous biomolecules such as the coenzymes: nicotinamide adenine dinucleotide (NADH) and flavin adenine dinucleotide (FAD) display fluorescent properties^(^^3^ ^,^^5^ ^)^. Fluorescent imaging is especially useful in the study of the nucleus or DNA as it is very difficult to observe DNA with traditional light microscopes. This can be remedied by staining it with Hoechst or DAPI, fluorescent chemicals specific in binding to DNA. The Hoechst stain is the preferred choice as it provides greater cell permeability and is less cytotoxic to cells compared to DAPI. It is excited by UV light (~350 nm) and emits blue light (~460 nm)^(^^3^ ^,^^6^ ^)^. Therefore, Hoechst is incredibly useful in studying nuclear morphology during apoptosis.

Apoptosis, referred to as programmed cell death, is a mechanism within multi-cellular organisms that occurs during embryonic development (formation of body parts). It controls the cell population, that is, by removing aged or unhealthy cells. It is also extremely important in destroying cells that have become infected or damaged by harmful agents, to prevent them from influencing the rest of the organism^(^^7^ ^)^. The morphological features of apoptosis entail cell and nuclei shrinkage (pyknosis), followed by the breakdown of nuclear material into smaller fragments (karyorrhexis). Finally, the organelles, DNA fragments, and cytoplasm are deposited into smaller membrane (apoptotic) bodies which break away from the main cell body (budding or blebbing) and are destroyed by the host’s immune cells^(^^7^ ^)^. Whilst fluorescence imaging is a powerful technique, the disadvantages of following these events in live cells are also prominent. Fluorescent nuclear stains including Hoechst, bind to the DNA and as a consequence prevent DNA replication during cell division, essentially ending the viability of the cells^(^^8^ ^)^. Phototoxicity is also increased during live cell fluorescence imaging. As the cells are exposed to high intensities of light, the fluorescent labels produce reactive oxygen species in their excited states, which in turn damages the structure and functionality of critical biomolecules^(^^9^ ^)^. Additionally, fluorophores can eventually become irreversibly damaged if they are not imaged correctly or for an extended period, referred to as photobleaching. It has been recommended to employ the optimum imaging parameters, spend minimum time searching for perfect images, and remove the oxygen from the medium. Regardless, all of these factors are still genuine limitations experienced by users of fluorescence microscopy^(^^10^ ^)^. Studies on apoptosis generally start with healthy cells imaged under optimal conditions. Such conditions need to be established first and could already introduce cell damage. The onset of the apoptotic process can then be induced by the addition of drugs or other stressors. Together with the time needed to prepare the samples (including repeats), which can be time-consuming, the whole process of fluorescence imaging can lead to the introduction of artifacts and ultimately failure of the experiment. To overcome these drawbacks, researchers have started to explore the latent information hidden in the bright-field images to simulate the fluorescent images using computer vision technologies. Up to now, there have been very few conventional methods for fluorescent image translation, the reason being that the amount of latent features within images is too big to be extracted manually, and it is even harder to align features between the two image domains.

The rapid evolution of machine-learning techniques provides new opportunities for applying image translation in this area. The first image translation method was implemented by Christiansen *et al.* in 2018^(^^11^ ^)^. They established a model based on deep learning that reliably predicts labels from transmitted-light images. Deep learning is a machine-learning technique that specifically uses deep neural networks^(^^12^ ^,^^13^ ^)^. In the field of image processing, it was first adopted for image classification and segmentation^(^^14^ ^,^^15^ ^)^. Deep convolutional neural networks (CNNs) are employed to extract useful and unapparent information from images automatically, circumventing the problems of manual feature extraction. The work of Christiansen *et al.* determined that there were strong relationships between bright-field images and the corresponding fluorescent images^(^^11^ ^)^. The idea of translating a bright-field image into a fluorescent image using deep learning is thus feasible.

**Figure 1.**
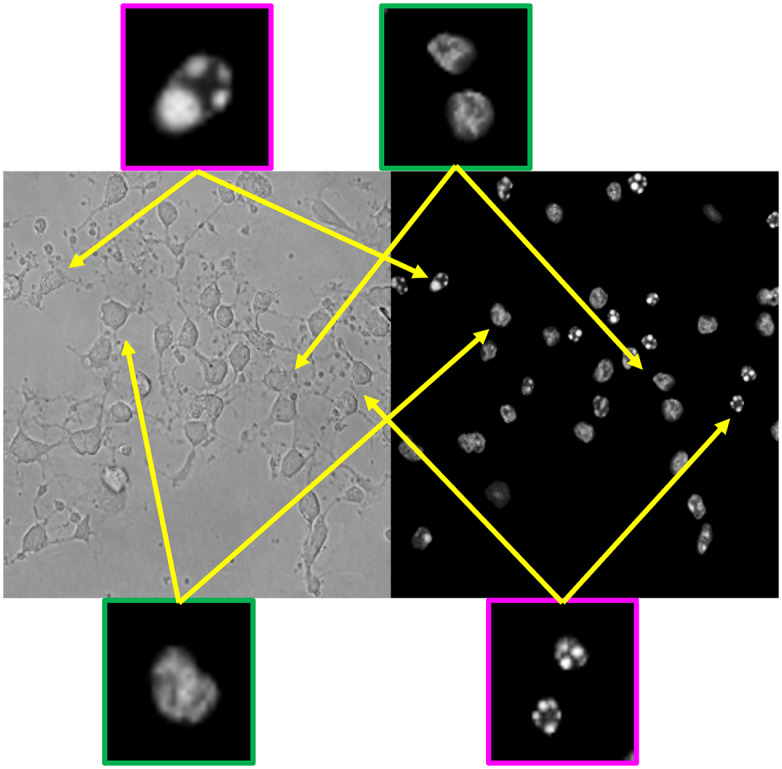
Information comparison between bright-field light microscopy (left) with fluorescence light microscopy (right) of the same cells. *Green* boxes indicate zoomed areas of healthy nuclei and *magenta* boxes indicate apoptotic nuclei with yellow arrows pointing to the position of the cells in the overview images.

The generative adversarial network (GAN) is one of the most popular neural networks and has been widely used in image translation tasks^(^^16^ ^–^^19^ ^)^. It has the advantage of solving the blurring problem that generally occurs when generating synthetic images^(^^20^ ^)^. GANs were recently introduced in fluorescent image translation and showed significant advantages in generating multiple channels of fluorescent images simultaneously and predicting sub-cellular structure details^(^^21^ ^–^^24^ ^)^. In some existing work, each channel of the output represents a specific labeling result with a different stain or dye derived from a single bright-field image^(^^21^ ^–^^23^ ^,^^25^ ^)^. For nuclei belonging to the same species but in different states, such as the instance shown in Figure 1, previous approaches would have difficulties in distinguishing between the different states of the nuclei, one such example of this is shown in Section 5. In this work, we propose a novel deep-learning model based on a conditional GAN (cGAN) aimed at translating bright-field images to fluorescent images with simultaneous nuclei health state inference. In addition, we extracted the spatial dependency information from the state inference process and used it as supplementary information for image generation. Inspired by self-attention mechanisms^(^^26^ ^,^^27^ ^)^, which have been used for calculating long-distance spatial dependencies during image generation, attention-based modules were included in our model for learning spatial dependencies. The attention-based modules take feature maps from both image generation and segmentation paths and feed them into the classification mechanism, a process referred to as cross-attention.

Following this general introduction to the subject, in Section 2, we set the scene by introducing previous work on fluorescent image translation using deep learning and in particular, approaches based on GANs. We also describe the attention network mechanism. In Section 3, we elaborate on the architecture of our model, including specific details of the generator and discriminator. Section 4 introduces the methodology, including the biological experiment, microscopy image acquisition, image pre-processing, and other preparation work for model training. Section 5 presents the experimental results. Section 6 is the ablation study. Finally, Section 7 concludes the article.

## 2. Theoretical Preliminaries

### 2.1. Image translation using convolutional neural network

In silico labeling (*ISL*) was one of the first published deep-learning approaches that could determine the correlation between transmitted-light microscopy images and fluorescent images^(^^28^ ^)^. It succeeded in predicting the location and intensity of nuclear labeling on bright-field images with good accuracy. The fluorescent images generated by the *ISL* model could be used in quantifying the dimensions of cellular structure and classifying the physiological state of cells^(^^11^ ^)^. Specifically, the *ISL* model performed the task of pixel-to-pixel classification and Christiansen *et al.* used a *z*-stack image, consisting of *z* slices of bright-field images, as the input for multi-channel fluorescent image prediction. The model was inspired by the popular U-Net^(^^29^ ^)^ and inception networks^(^^30^ ^)^. For each translation, five image patches were cropped out of the input bright-field image stack with different side lengths at the same centroid location and simultaneously fed into five independent input computational heads of the model, respectively; the outputs from the five computational heads were concatenated together and transmitted to the final prediction computational head of model to synthesize the fluorescent images. As each input computational head processed one side-length area, it allowed the model to learn spatial information from multiple spatial extents. The other special point of this network was that it was composed of repeated modules; the mutually independent modules had the same architecture but different parameters that controlled the number of hidden convolutional kernels. The *ISL* model implemented the translation piece by piece. A stack of bright-field image patches with 
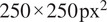
 was fed into the model. The *ISL* model predicted an 8-bit gray-scale image, where each unique gray-scale value had a probability. For each pixel, these probabilities were represented by a 256-element vector. Such a process ensured accuracy; however, it sacrificed computational efficiency, as the model was large and the parameters for each repeated module were hard to tune^(^^11^ ^)^.

Soon after the first trial for fluorescent image translation, Ounkomol *et al.* ^(^^22^ ^)^ proposed their work for label-free prediction of multi-channel immuno-fluorescence (IF) images from 3D transmitted-light or 2D electron microscopy (EM) images. As EM images contain plenty of sub-cellular structure information, this model was capable of presenting more intracellular details. The model can also automatically register EM images on large target IF images. The model for label-free prediction was a CNN based on the U-Net architecture. It contained down- and up-sampling substructures with shape concatenation processes. Compared to an *ISL* model, its architecture was much more intuitional and close to the initial U-Net architecture^(^^22^ ^)^.

### 2.2. Image translation based on generative adversarial neural network

Image translation approaches with deep learning have gone through a breathtaking evolution in recent years. The initial attempt using CNNs was an impressive achievement, but its results tended to be blurry. This was because the loss function was minimized by averaging all plausible outputs^(^^20^ ^)^. To overcome the disadvantages of the blurring issue, the generative adversarial neural network (GAN) was proposed^(^^16^ ^,^^31^ ^–^^33^ ^)^. The basic concept for GAN was adding another CNN to the generative model for discriminating whether the output is real or fake. This adversary system was trained together and encouraged the generative network to produce more convincing results and prevent the outputs from being blurry.

The original GANs generated outputs from random vectors^(^^34^ ^,^^35^ ^)^; however, subsequent variants have since been derived. For example, Li *et al.* employed CycleGAN^(^^18^ ^)^ for unsupervised content-preserving transformation for optical microscopy (UTOM)^(^^21^ ^)^. CycleGAN employed two generators to transform images between two domains back and forth, respectively, and two discriminators were used for penalizing whether the generated images belong to each domain; images transformed forward and then transformed backward should be the same. One speciality of CycleGAN was that images from the two domains did not need to be corresponding, so it allowed for unsupervised translation^(^^18^ ^)^. Meanwhile, the UTOM model used a saliency constraint to maintain the saliency within the images unchanged during transformation to avoid distortion of content. Generators in UTOM had the same number of input and output channels to simplify the transactions. It was shown that UTOM achieved comparable performance to the supervised CNN model without having any paired training datasets. However, the training dataset for UTOM was larger than the supervised model, otherwise, the accuracy of translation would be limited^(^^21^ ^)^.

UTOM achieved a great performance for image translation, but the limitation of the dataset could not be neglected. Therefore, a model that combined the advantages of supervised learning and GANs could be another solution. cGAN was a promising approach suitable for tasks with limited but paired datasets^(^^36^ ^)^. The discriminator in cGANs not only judged whether the outputs were real or fake but also determined whether the inputs and outputs were consistent. As a supervised learning process, the loss of the generator was a combination of the discriminating result and the distance between the outputs and ground truth. Isola *et al.* ^(^^20^ ^)^ proposed an image-to-image translation model with cGAN. The model succeeded in image generation from sparse annotations, future frame prediction, or photo reconstruction. Their generator for cGANs was inspired by U-Net^(^^29^ ^)^, and the discriminator used a specially designed “PatchGAN” classifier to enhance the spatial penalization^(^^20^ ^,^^37^ ^)^. This approach was widely used as the benchmark for style transfer of cellular images^(^^38^ ^)^.

Based on the concept of cGAN, Han *et al.* ^(^^39^ ^)^ produced a model for cellular image translation. The model was established for the DIC to PC transition. The only difference in the architecture from the original cGAN model was the model took the predefined cell masks as additional inputs and the system consisted of two discriminators, one for DIC–PC pairs and one for mask–PC pairs. This model showed that the generator performed better compared to only DIC images for inputs.

After the success of applying cGANs in microscopy image translation, Nguyen *et al.* ^(^^23^ ^)^ designed a complex system with multiple cGANs applied on microscopy images taken from breast-cancer and bone-osteosarcoma cell lines. The system implemented not only the transformation of images from fluorescent images to PC images but also the interpretation between different fluorescent channels. The architectures for each translation model were basic pixel-to-pixel image translation networks introduced in the work of Isola *et al.* ^(^^20^ ^)^, and they implemented a novel algorithm for evaluating the generated result. Their work achieved great success in the prediction of subcellular localization of proteins with other proteins on well-registered images. It proved that a cellular image translator based on cGAN was an effective tool for studies about relationships between proteins and organelles and to visualize the subcellular structure^(^^23^ ^)^.

Lee *et al.* provided the DeepHCS++ model for the fluorescent image translation task^(^^40^ ^)^. The architecture of the DeepHCS++ model consisted of two parts: transformation and refinement networks. The transformation network was similar to the label-free model of Ounkomol *et al.*; however, instead of using one up-sampling decoder for multi-task learning, three independent decoders were applied to produce three channels of fluorescent images, respectively. The intermediately produced fluorescent images were then fed into three U-Net shape refinement networks for the final predictions. Conditional adversarial loss calculations were only applied to the final output images rather than intermediate products. Results showed that the performance for translation-refinement networks was better than single translation networks^(^^40^ ^)^.

### 2.3. Attention neural network

Images generated by GANs derived from lower-resolution feature maps and higher-level details are calculated with spatially local points. This process has a significant limitation as it only takes spatially local information for calculations^(^^27^ ^,^^41^ ^)^. However, the *ISL* model showed that the translation required information from multiple spatial extents of correlated bright-field images, which is why it took five scales of inputs. Fortunately, self-attention GANs (SAGANs) provided an approach for long-range dependency modeling for image generation.

Self-attention mechanisms allowed for computing dependencies within a single sequence without the restriction of position^(^^26^ ^,^^42^ ^,^^43^ ^)^. It had been successfully used in sequence-to-sequence processing such as reading comprehension, textual analysis and language translation. This approach can be transferred to spatial analysis in image processing^(^^44^ ^)^. Zhang *et al.* proposed the SAGAN, which aimed to calculate the dependencies of one position on all others through feature maps. The result showed that the SAGAN was an effective model for the calculation of long-range dependencies and upgraded the qualities of output images^(^^27^ ^)^. Besides, SAGANs applied spectral normalization^(^^45^ ^–^^47^ ^)^ to the generator and discriminator to enhance the stability of GAN training. The mechanisms of SAGAN were introduced in our model, further details will be elaborated in the next section.

## 3. Proposed Approach

The framework proposed in the translation task relies on a cGAN. Similar to the basic GAN network, it also includes a conditional generator system for image translation, in conjunction with a discriminator to evaluate its outputs. Both generator and discriminator are composed of a series of sub-modules. What is different is that the generator contains two generation paths: one for image translation and one for nuclei segmentation. Thereinto, nuclei segmentation is implemented by producing masks for nuclei with different health states. Attention modules in the network are used for transferring information between the two generating paths within the generator. The module is based on self-attention mechanisms, and we refer to it as the cross-attention module. Because of the dual-output generator, the loss function consists of a weighted combination of both paths.

### 3.1. Generator

**Figure 2.**
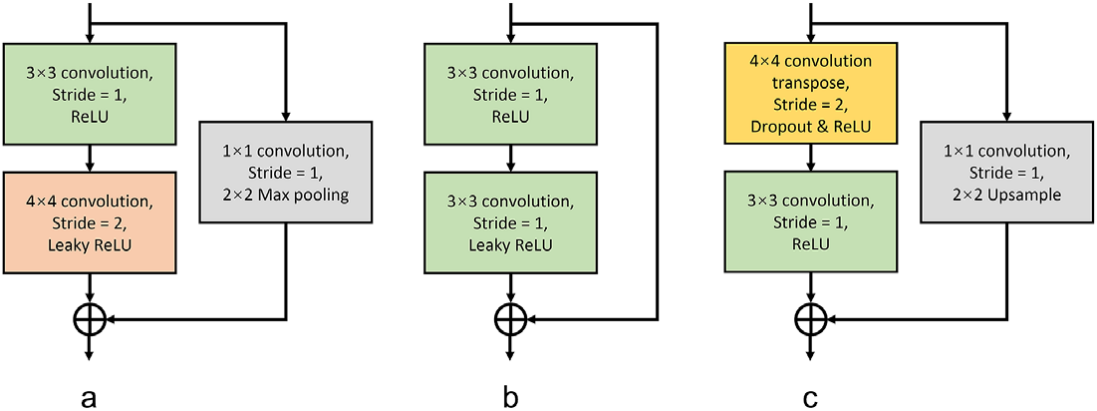
Network architectures of sub-modules for generator. (a) Down-sampling sub-module. (b) Bottleneck sub-module. (c) Up-sampling sub-module.

**Figure 3.**
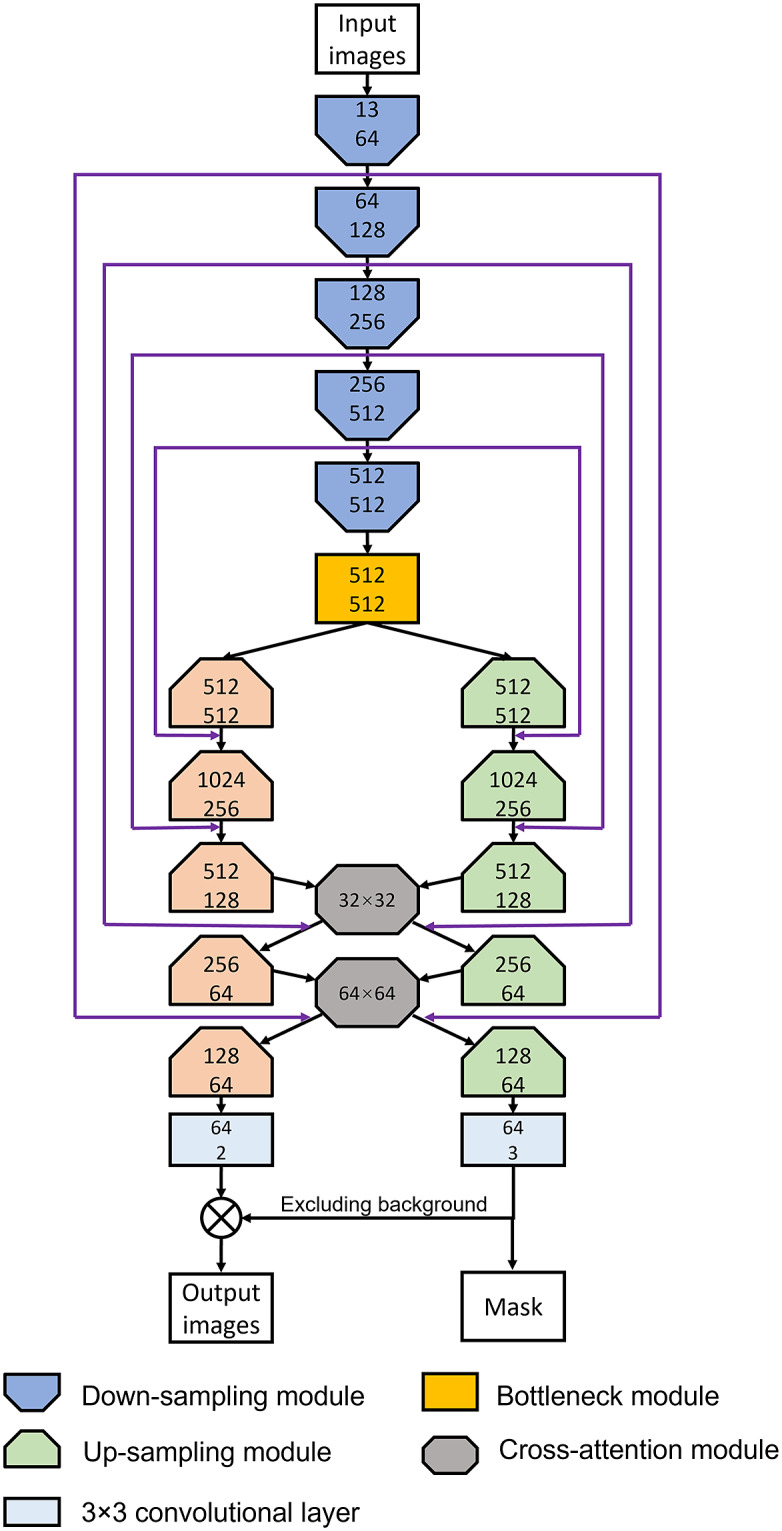
Generator architecture. The numbers in each sub-module indicate the numbers of input and output channels, respectively. Architectures of sub-modules are presented in Figure 2, and attention modules are shown in Figure 4.

The architecture of the generator is inspired by the U-Net^(^^29^ ^)^ and ResNet^(^^48^ ^)^ networks. U-Net was the first network designed for biological image segmentation. It contains an encoder–decoder system linked by skip connections. The encoder consists of a down-sampling process to transform the images to feature maps with more channels but smaller sizes; on the contrary, the decoder is an up-sampling process that transforms the intermediate feature maps to the output size. Intermediate outputs from the encoder are concatenated to the decoder which enhances spatial accuracy. One difference in our model is its two independent generation paths for image translation and nuclei semantic segmentation. Each channel of the image translation output represents one health state of the nuclei, meanwhile, the nuclei segmentation path provides binary masks for each state or background. The generator is constituted by a series of sub-modules, and each sub-module corresponds to one down-sampling or up-sampling process. Additionally, there is one bottleneck module to connect the encoder and decoders. Figure 2 shows the structure of the sub-modules. Skip connections for residual learning are employed in the sub-modules to enhance the robustness of the model. Each sub-module contains three parts, a shape-invariant convolutional layer, a sampling convolutional layer, and a residual connection^(^^48^ ^)^. The final activation function layer for the image generation decoder is Tanh to restrict the range of image values between −1 and 1. The output of the mask generation process is sent into a SoftMax function to calculate the probability of pixels belonging to each category. The output of the mask generation path has one more channel than the image generation path, which is for the background probability prediction. Finally, the image generation outputs are multiplied by the probability results from the segmentation path to produce the translation result. Details of the architecture of the generator are shown in Figure 3.

### 3.2. Attention-based module

**Figure 4.**
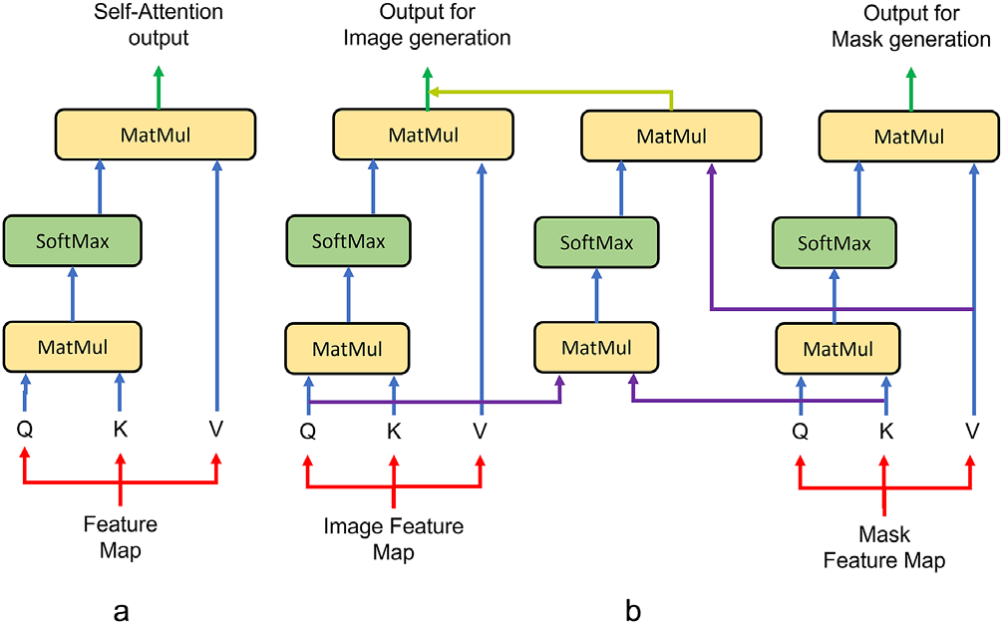
Attention module architectures. (a) Architecture of the self-attention module. (b) Architecture of the cross-attention module.

Attention algorithm-based modules are utilized to learn long-distance spatial dependencies. They are inserted between the decoders to exchange spatial information during image generation. The initial attention algorithm module is self-attention which calculates the spatial dependencies within the feature maps themselves. However, we want the attention module to also share the spatial dependencies between two generation paths. As our attention module derives from the self-attention module, we will describe the structure of the self-attention module first, and then compare the improvement of our attention-based module. The self-attention network is presented in Figure 4a, and the processing steps are listed below.

1. Send the input feature map stack into three 1 × 1 convolutional layers and reshape the outputs into 1D sequences, 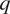 , 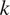 , and 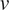 .
2. Calculate the attention of feature 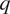 and 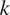 .
3. Feed the attention output into a SoftMax activation function.
4. Calculate the attention of the SoftMax output with feature 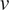 .
5. Reshape the 1D sequence back to the original shape.
6. Send the feature into another 1 × 1 convolutional layer to keep the self-attention module output the same shape as the input.
7. Add the self-attention output back to the feature map stack and multiply with a learnable parameter.

In our model, our aim is to share the spatial dependency information from both generation paths. Therefore, we extract hidden features from both generation paths using in Step 1, ( 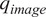 , 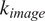 , and 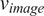 from image feature domain; 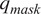 , 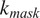 , and 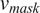 from mask feature domain). Our attention module contains two self-attention calculation paths which remain the same as the self-attention module; at the same time, hidden feature 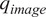 (from the image path), 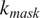 and 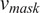 (from the mask path) are sent to the algorithm from Step 2 to Step 6 to measure the correlation between decoders. This process is similar to the work in by Hou *et al.* ^(^^49^ ^)^. These are concatenated with the image self-attention outputs and sent to the next layer in the image generation path. As previous work proves that a U-Net with one encoder–decoder pair is enough for biomedical image semantic segmentation^(^^29^ ^)^, we decided not to make the mask generation process as complex as image generation. Therefore, the correlated attention algorithm is not applied to the mask generation path. The module is named cross-attention as it takes inputs from both paths and feeds them back. The cross-attention modules are inserted between feature map layers of the same size from different generation decoders. The architecture of the cross-attention module is presented in Figure 4b.

### 3.3. Discriminator and spectral normalization

**Figure 5.**
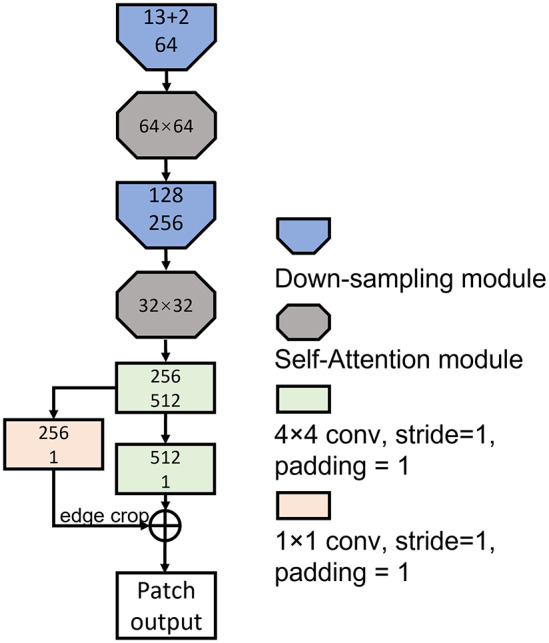
Discriminator architecture. The numbers in each sub-module indicate the numbers of input and output channels, respectively. Numbers in attention boxes represent the size of input feature maps.

The discriminator does not produce translation results but instead guides the training destination of the generator and self-upgrades simultaneously. Normally, the discriminator is a down-sample process which assesses whether the input is good or not. Isola *et al.* introduced a discriminator named PatchGAN for their image-to-image translation network^(^^20^ ^)^. PatchGAN outputs *N* × *N* patches and penalizes the patches independently, where the size of patches can be much smaller than the full size of the image. They found that the PatchGAN in the discriminator performed well in enforcing correctness at high frequencies and that low-frequency correctness could be achieved using conventional methods, such as the L1 distance restrictor. The discriminator takes the concatenation of bright-field image stacks and generated translated images as the input and runs a series of convolutional calculations. The architecture of the discriminator is shown in Figure 5; it is also made up of down-sampling sub-modules which have a similar structure as the one used in the encoders of the generator. Self-attention modules are applied in our discriminator; they are inserted after the down-sampling sub-modules at higher levels.

To improve the performance of the discriminator, Miyato *et al.* created a new normalization method called spectral normalization and applied it to the discriminator of their GAN^(^^45^ ^)^. They discovered that spectral normalization can stabilize the training of the discriminator, as it tuned fewer extra hyper-parameters and had lower computational costs compared to other regularization techniques. In the work of Zhang *et al.*, spectral normalization was applied to both the generator and discriminator. This achieved better performance compared to applying it only to the discriminator. Hence, we also apply spectral normalization to our discriminator and generator^(^^27^ ^)^.

### 3.4. Loss function and evaluation metrics

The fundamental working mechanism of GANs can be understood as a competition between the generator and discriminator. The generator aims to produce outputs that fool the discriminator into being unable to distinguish whether its products are real or fake. On the contrary, the discriminator aims to train itself to not be fooled by the generator. Mathematically, this process can be regarded as finding the solution to minimum and maximum objective functions. In cGAN, the generation process is under given conditions, in the translation task the output fluorescent images need to be correlated to the input bright-field image stacks. Therefore the discriminator needs to take the input bright-field image stacks as the condition during the penalization. The objective function of cGAN is shown in Equation (1):
(1)

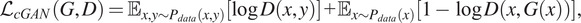

where 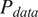 is the ground truth dataset, 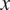 is the input bright-field image, and 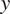 is the target fluorescent image; 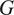 and 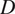 represent the generator and the discriminator, respectively. The generator is trained to minimize the objective function and the discriminator is trained to maximize it; the generator is described in Equation (2):
(2)

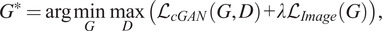

where 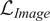 is the conventional loss between the generated outputs and the ground truth targets, such as L1 loss (details to follow). 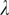 is the weight that balances the two types of losses.

The total loss for the system is a combination of adversarial loss and conventional losses. Typical conventional losses include mean absolute error loss (MAE) or mean squared error (MSE) loss, also known as L1 and L2 distances, respectively. The conventional loss function does well in penalizing the low-frequency errors which are supplementary to adversarial loss. In previously published approaches^(^^20^ ^,^^40^ ^)^, the weight of the conventional loss is normally one or two orders of magnitude higher than the adversarial loss in cGAN, we will follow this precedent. Meanwhile, MAE losses are used rather than MSE losses in image generation as research showed that MAE-based methods cause less blurring in practice^(^^20^ ^)^. Moreover, based on the experience from Hore *et al.*, we introduce a structural similarity index measure (SSIM) distance for the conventional loss calculation^(^^40^ ^)^. SSIM is a method for measuring the similarity between two images; it is a perception-based model that considers the degradation in structural information, which is expressed in Equation (3):
(3)

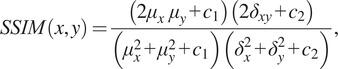

where 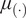 and 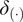 are the mean and variance of 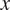 and 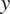 , 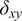 is the covariance of 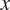 and 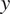 ; 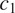 and 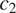 are constant values based on the intensity range of 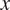 and 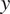 . The output value of the SSIM function is from 0 to 1, the more similar they are the higher the value is, and hence, the SSIM distance of 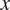 and 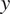 can be calculated as 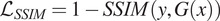 . Thus, the conventional loss for image generation is presented in Equation (4):
(4)

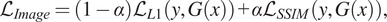

where 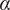 is the weight to balance L1 loss and the SSIM distance, 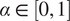 .

Other than losses from the image generation path, the output of the mask generation path predicts the possibilities of pixels belonging to each category. The loss of segmentation from the mask generation is defined as the classical cross-entropy. The final loss for the generator is a weighted sum of image generation losses, including conventional loss and adversarial, and mask loss, total loss calculation is shown in Equation (5):
(5)

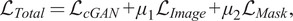

where, 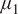 and 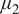 are weights for each loss. In practice, we chose a dynamic weight for 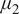 , the loss for mask generation. A higher value for 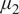 was applied to ensure the mask generation reaches the convergence point first so that the mask generation path provides highly credible spatial information to the cross-attention module, and gradually lowers the weight of the mask to enhance the image generation.

In addition, the performance of the model is evaluated using three metrics: the L1 distance (i.e., the MAE), the structural similarity index (SSIM), and the standard peak signal-to-noise ratio (PSNR)^(^^50^ ^)^.

## 4. Experimental Setup

### 4.1. Biological experiment and microscopy operation

In this section, we further describe the methodological approach from a biological point of view, providing further motivation for the work and presenting the datasets employed.

The cells used in this study are Chinese hamster ovary (CHO)-K1 cells [ATCC-CCL-61], which are induced to undergo apoptosis, to analyze and predict the state of their nuclei. These are cultured in Ham’s F-12K (Kaighn’s) Medium (Life Technologies Limited, Gibco, Paisley, Scotland, United Kingdom) supplemented with 10% fetal bovine serum (FBS). They are grown in T-75 flasks (10 ml of culture medium) and incubated at 37°C and 5% CO_2_.

1. When the cells have reached minimum confluency of 60%, ~100,000 cells are seeded onto glass coverslips (13 mm diameter) in 500 μL of medium and left to adjust overnight. All rinsing steps are done with Phosphate buffered saline (PBS).
2. To induce apoptosis, the cells are exposed to 500 μL of 1 μM of staurosporine (in medium, Apexbio Technology LLC, Houston, Texas, United States) and incubated for 0, 1, 3, 6, and 8 hr.
3. Once the respective time has been reached, the cells are fixed in 4% Paraformaldehyde (PFA) in PBS for 30 min.
4. The nuclei are stained with Hoechst solution (1:10,000 dilution in medium, Thermofisher Scientific, Pierce Biotechnology, Rockford, Illinois, United States) for 10 min.
5. Finally, the coverslips are mounted onto glass slides and left to curate overnight.

The cells are imaged on a Leica SP5 confocal laser scanning microscope using an HC PL APO CS2 63×, 1.4 NA oil objective. Hoechst-stained nuclei are excited with a 405 (nm) diode laser (laser power: 7%). Single-plane bright-field images and fluorescent image stacks are taken of the nuclei. The fluorescent image stacks have a *z*-axis range of 7.2 μm and consist of 24 slices spaced 0.3 μm apart.

### 4.2. Dataset preparation

**Figure 6.**
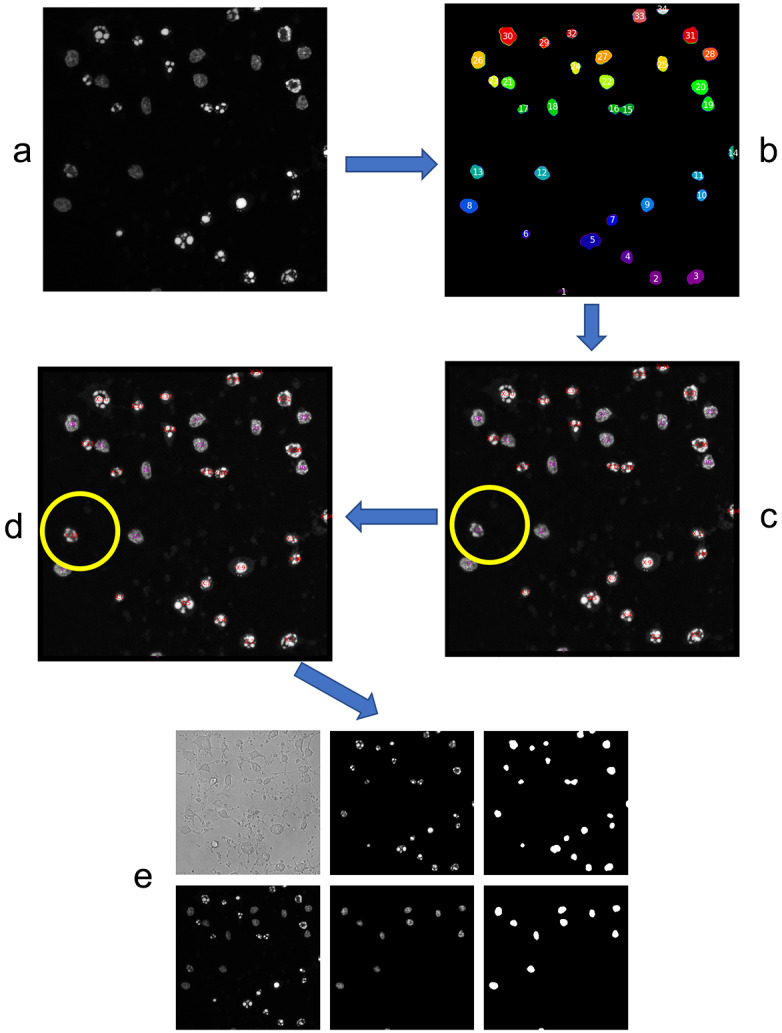
Dataset preparation process. (a) Maximum intensity along *z*-stack of fluorescent images, (b) threshold and watershed segmentation output, (c) automatic classification result, (d) manually revised result, the revised individual in the yellow circle, (e) example of image dataset for training, contains bright-field images, split fluorescent images, and masks.

Our method is designed to produce fluorescent images together with semantic segmentation. The most important part of the image pre-processing pipeline was segmenting the fluorescent images based on the health state of each nucleus. The states of the nuclei change over time, that is, after 3 hr nuclei start to split. In the pre-processing phase, fragmented nuclei were merged using Gaussian and median filters. Subsequently, a marker-controlled watershed segmentation process was applied to separate adherent nuclei. All steps in the pre-processing pipeline are listed below and shown in Figure 6.

1. Resize raw images to an appropriate size that maintains adequate information. In this work, we resize the images from 
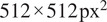
 to 
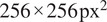
 .
2. Apply min–max normalization for each raw fluorescent image and rescale to 0 to 255 (8-bit intensity range).
3. Concatenate fluorescent images along *z*-stack. Choose the maximum value along the *z*-stack for each pixel and generate the maximum image.
4. Apply contrast limited adaptive histogram equalization (CLAHE)^(^^51^ ^)^ to each image. Set the contrast limiting value to be 2, and the size of tiles to be 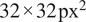 .
5. Apply Gaussian smoothing to images using a 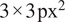 kernel. In practice, we apply it four times so that fragmented spots from the same nuclei are connected.
6. Apply median filtering with a 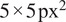 kernel. In practice, we apply it 12 times to remove salt-and-pepper noise from the images.
7. Calculate the Otsu threshold for the images and generate the mask for nuclei.
8. Calculate the distance for pixels within the mask to the mask boundaries. Then select the local maximum points on the distance maps and label them as the kernel for individuals.
9. Apply the watershed algorithm^(^^52^ ^,^^53^ ^)^ to segment the masks.
10. For nuclei incubated in staurosporine for less than 8 hr, the number of apoptotic cells is much less than healthy cells. The split nuclei can be easily filtered out manually.
11. For nuclei at 8 hr. Apply individual segmented nucleus masks to the maximum fluorescent images. Calculate the standard deviation of the intensity value within each segmented nucleus image. Use the Otsu thresholding method^(^^54^ ^)^ to separate these nuclei into two groups. The group which has a higher value of standard deviation corresponds to fragmented nuclei and to intact nuclei. Inspect the output of the segmentation process based on standard deviation, and manually correct the splitting results.
12. Individual masks belonging to the same group are added together and used to generate the masks for healthy nuclei and apoptotic nuclei.
13. Apply both nuclei masks on the center layer of the *z*-stack fluorescent images after CLAHE. Each fluorescent image is divided into two channels. The two-channel fluorescent images are used as the ground-truth targets for training.
14. For bright-field images, apply min–max normalization for rescaling and concatenation along *z*-stack.

### 4.3. Dataset augmentation

Due to the limited number of images in our dataset, data augmentation was applied for the training of the model. Random flips and rotations were applied, followed by a random crop of 
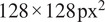
 out of 
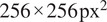
 . Random flips and rotations encourage the model to not be restricted by the orientation of the input images. A random crop of images helps individual nuclei maintain the same sizes and ratios (width to height) compared to the original images, but the locations of nuclei on images vary at each iteration. Data augmentation ensures the inputs to the model are different for every epoch and prevents over-fitting of the model.

### 4.4. Training details

The code was implemented using Python and PyTorch. This work was carried out using the BlueCrystal Phase 4 facility of the Advanced Computing Research Centre, University of Bristol (http://www.bristol.ac.uk/acrc/). The system is equipped with Nvidia P100 GPU with 16 GB of RAM. The optimizer for the generator and discriminator used here was the Adam optimizer with beta values of 0.5 to 0.999^(^^55^ ^)^, while the learning rates were 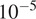 and 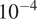 , respectively^(^^56^ ^)^. The batch size for training was 8. The total number of learning epochs was 4,500. Cross-validation was applied to the training dataset, with the number of images for training, validation, and testing being 66:12:8. The training and validation datasets were rearranged every 50 epochs. As mentioned above, the weight for the mask generation loss varies during each iteration; the initial weight was set to 250 and decayed 10% every 1,500 epochs and no lower than 2.5.

## 5. Results

In our model, whose architecture is shown in Figure 3, there are five up-sampling layers. Therefore, there are four intervals between adjacent up-sampling layers for inserting self-attention modules, so theoretically, there should be 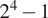 models to be tested. We used four-digit binary numbers to indicate the locations of the attention modules. Zhang *et al.* found the attention level applied at feature maps with larger sizes received better performance^(^^27^ ^)^. Therefore, in our strategy of choosing the insertion sites, we filled in lower intervals (where feature maps had larger sizes). As such, we selected three models for performance evaluation and comparison: 0001 (attention module only appeared at the last interval), 0011 (last two intervals), and 0111 (last three intervals). We also selected two models as references. The first was the cross-attention cGAN (XAcGAN) model, but with no cross-attention module applied; this was labeled as 0000. The second was the original image translation model using cGAN, the pixel-to-pixel image translation model, introduced in Isola *et al.*’s work^(^^20^ ^)^. The second reference model had six down-sampling layers and six up-sampling layers with skip connections. This second reference model had no residual network module or spectral normalization in either the generator or discriminator.

**Figure 7.**
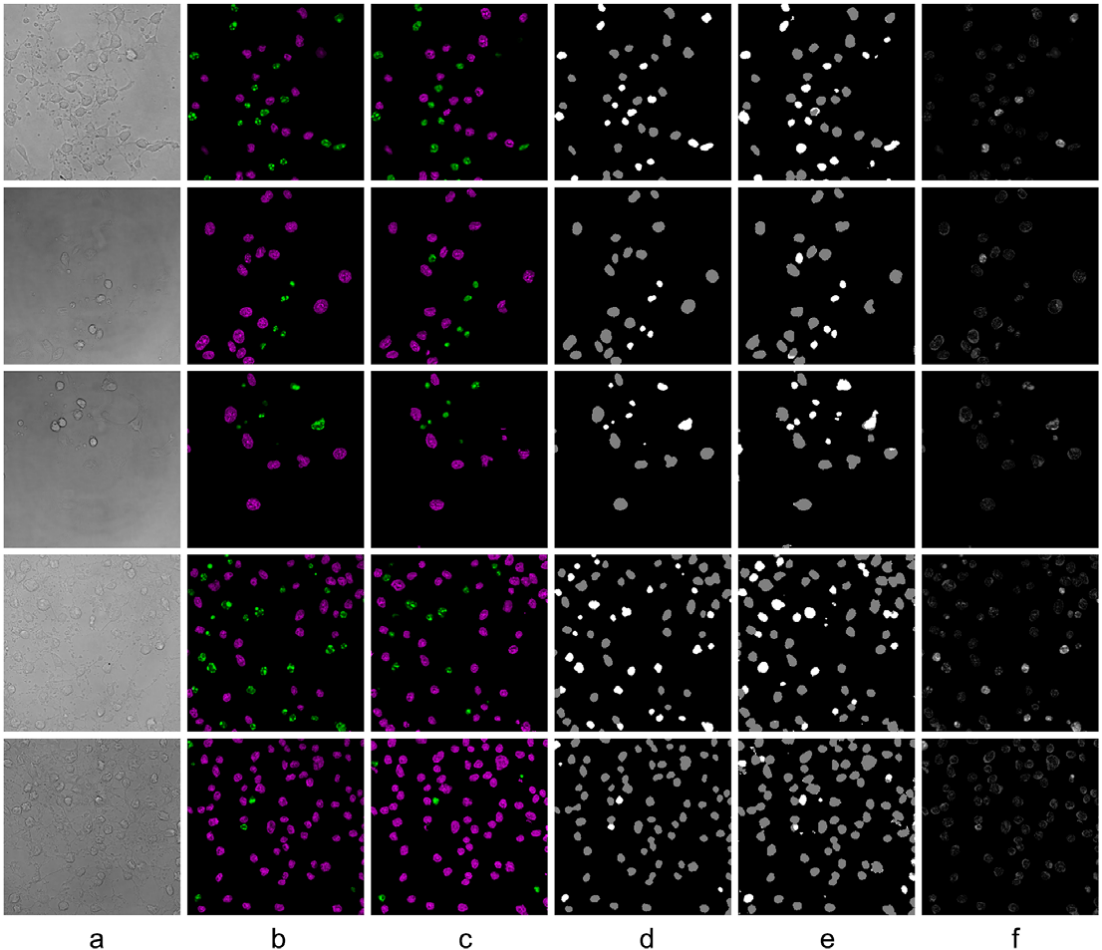
Translation results of cross-attention cGAN (XAcGAN) model with attention module location “0011.” Column (a): middle slices of input bright-field image stacks; column (b): ground truth fluorescent images, with nuclei false-colored such that magenta represents healthy nuclei and green represents apoptotic nuclei; column (c): translation results from the model with equivalent false-coloring applied; column (d): the ground truth classification of nuclei, gray represents healthy nuclei and white represents apoptotic nuclei; column (e): the semantic segmentation results by XAcGAN 0011 model; column (f): the MAE error maps between the target and generative fluorescent images.

The prediction results for XAcGAN models are listed in Table 1 together with the pixel-to-pixel model. As expected, the XAcGAN models’ performances were significantly better than the pixel-to-pixel model in all evaluation metrics. Of the three tested models, model 0011 received the best scores, which are highlighted in bold. Figure 7 shows the prediction from the XAcGAN 0011 model.

**Table 1.** Performance of cross-attention conditional GAN model.

		Cross-attention module inserted location^a^			
Evaluation metrics	Pixel-to-pixel	0000	0001	0011	0111
L1_distance	0.0546	0.0320	0.0332	**0.0256**	0.0303
PSNR	20.5644	22.0651	21.8404	**23.6517**	22.2572
SSIM	0.8494	0.9132	0.9145	**0.9310**	0.9149

Abbreviations: PSNR, peak signal-to-noise ratio; SSIM, structural similarity index measure.

a The inserted location is presented by a four-digit binary number, the first digit represents the position between the first and second down-sampling layers and can deduce the rest like this. Digit “1” means an attention-based module is inserted at this position, and vice versa.

### 5.1. Contribution of attention-based module

**Figure 8.**
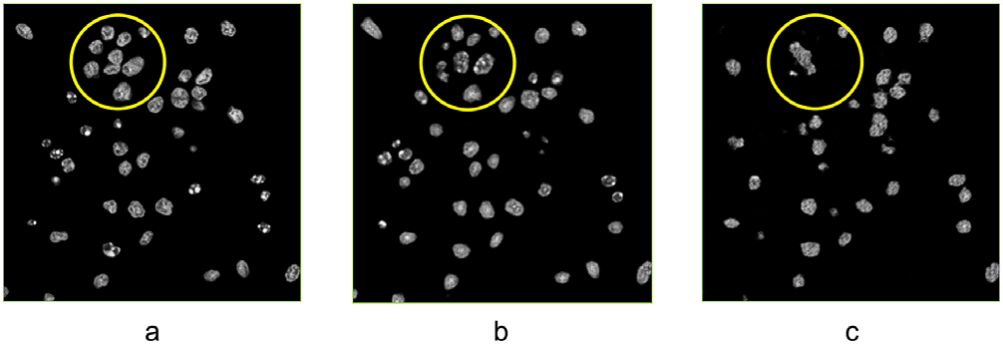
Detection accuracy comparison. (a) Ground-truth fluorescent image, (b) XAcGAN model result, (c) pixel-to-pixel model result. Translation result from the XAcGAN model has a higher accuracy of nuclei detection than the non-attention model.

Compared to the pixel-to-pixel model, the attention-based model had higher accuracy in detecting nuclei. This proves that the attention element contributed to the long-distance dependency analysis when detecting nuclei from cell images (see Figure 8). Cells took up larger areas on bright-field images than nuclei in fluorescent images and included latent information on the nuclei’s state of health. However, without an attention-based mechanism, this information was restricted because of the sizes of convolutional kernels, which failed to transfer them to higher-level feature maps. To promote accuracy in nuclei detection, the attention-based module from the XAcGAN model played a vital role.

### 5.2. XAcGAN model for necrotic cell detection

**Figure 9.**
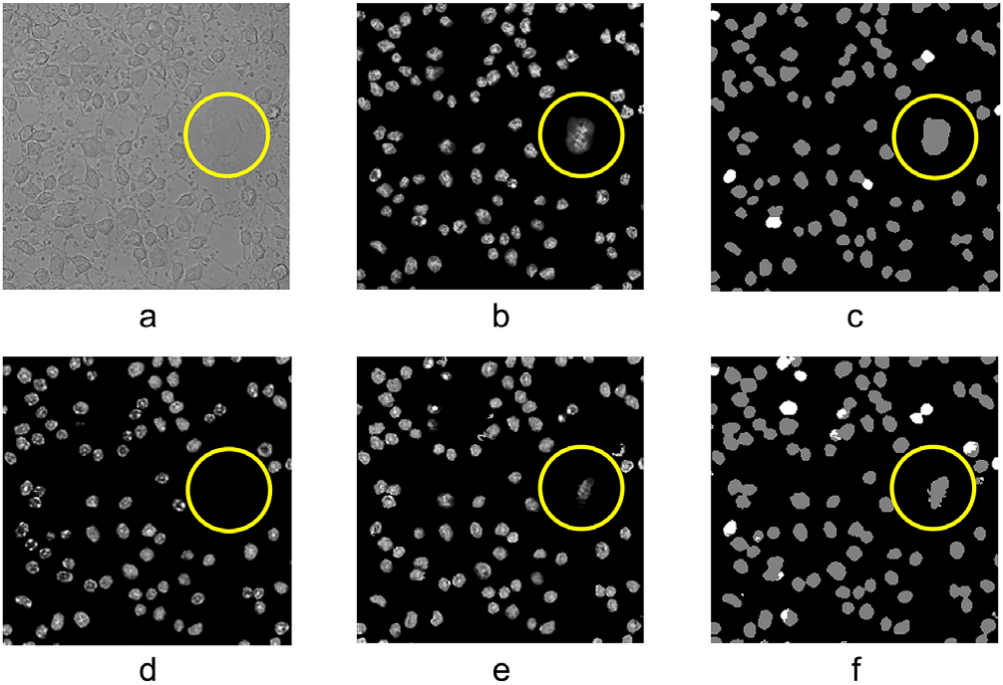
Translation result of the necrotic cell. (a) Bright-field image. (b) Ground-truth fluorescent image. (c) Ground-truth nuclei classification result. (d) Result from the model without cross-attention module. (e) Result from XAcGAN model. (f) Nuclei segmentation result from XAcGAN model. Yellow circles indicate the necrotic cell under karyolysis.

The cross-attention model has advantages in finding necrotic cells which are inconspicuous in bright-field images and out-of-focus in fluorescent images. Apoptosis is described as an energy-dependent process which is coordinated by cysteine-aspartic proteases called caspases, while necrosis is referred to as uncontrolled cell death^(^^57^ ^)^. Necrosis is an energy-independent process and is a consequence of severe and sudden cellular damage to the extent that the cell is no longer functional. A notable difference in necrosis is the morphology of the nuclei. Within necrotic cells the nuclei undergo karyolysis when the nuclear material dissolves in the cytoplasm (broken down by endonucleases)^(^^7^ ^,^^58^ ^)^, which is observed in Figure 9. In Figure 9, we can see that the reference model failed to recognize the necrotic cells, but the cross-attention model found it, though the area of the target predicted by the cross-attention cGAN model was smaller than expected. This is an illustration of the contribution of the mask generation path to image translation. On the input image stacks, the necrotic cells were easily overlooked as the intensity changes in this area were not distinct. The mask generation path had better performance in indicating the location of these blurry items and leading the image generation path to predicting intensity.

### 5.3. Influence of the number of slices of bright-field image stack

We verified the performance of the XAcGAN model with a different number of input bright-field image slices. In the work of Christiansen *et al.* ^(^^11^ ^)^, they found if the number of input slices was higher than five, the performance of the model did not improve too much. We performed a similar test on our model, using 1, 3, 5, and 7 slices. An odd number of slices was used for all tests since this maintained the same middle layer for each image stack. The input bright-field stack had 13 slices, the space distance of adjacent slices was 0.3 μm and the total depth of the stack was 3.6 μm. For input images with 3, 5, and 7 slices, the test was applied using two approaches. In the first, inter-slice separations were kept at 0.3 μm, thus yielding stack depths of 0.6 μm, 1.2 μm, and 1.8 μm, respectively. In the second test, slice separations of 1.8 μm, 0.9 μm, and 0.6 μm were used to produce fixed stack depths of 3.6 μm. The results of the tests are shown in Table 2.

**Table 2.** Performance of cross-attention cGAN (XAcGAN) model.

Input slice(s)	1	3		5		7	
Separation (μm)	n/a	0.3	1.8	0.3	0.9	0.3	0.6
L1_distance	0.0455	0.0475	0.0411	0.0355	0.0317	0.0359	0.0326
PSNR	19.3453	19.0231	20.3605	21.2040	22.2555	21.0891	22.0951
SSIM	0.8878	0.8866	0.9037	0.9043	0.9095	0.9102	0.9084

Abbreviations: PSNR, peak signal-to-noise ratio; SSIM, structural similarity index measure.

**Figure 10.**
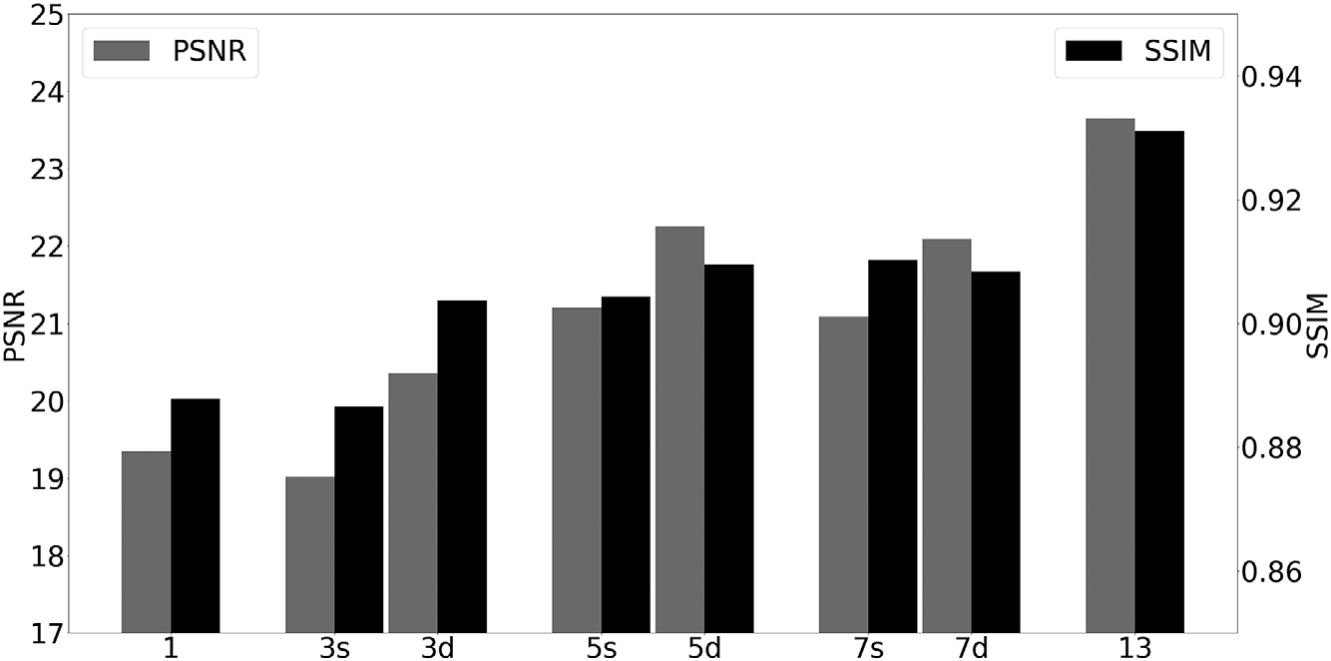
Performance of different numbers of input slices of bright-field image stacks, the numbers at the bottom indicate the number of image slices, “s” means slice separation remains unchanged, and “d” represents total depth unchanged.

From Table 2, we can see that the input image with only one slice had the worst performance. Undoubtedly, the more slices the bright-field image stack had, the better result obtained. Meanwhile, the depth of the bright-field image stack had more influence on the translation result. For instance, from Figure 10, for a stack with three slices, performance for stack depth 3.6 μm and slice-separation 1.8 μm was significantly better than for stack depth 0.6 μm and slice-separation 0.3 μm. The reason for this could be the closer slices contained less 3D space information and led to poorer performance. As the number of slices of the input image stack increased, the improvement of the translation result was limited.

## 6. Ablation Study

The cross-attention module was inspired by the self-attention module from the SAGAN model^(^^27^ ^)^. Therefore, we compared the performance of these two modules. As the self-attention module took one stack of feature maps as input, the self-attention cGAN (SAcGAN) model for fluorescent image translation contained only one independent generation path. We first applied the SAcGAN model only for translating fluorescent images without outputting the nuclei state evaluation. In this case, the model had one output channel and nuclei with different states were presented in one image. We then tried to train the SAcGAN model with two output channels aimed to test the performance of nuclei classification without the help of a mask generation path.

**Figure 11.**
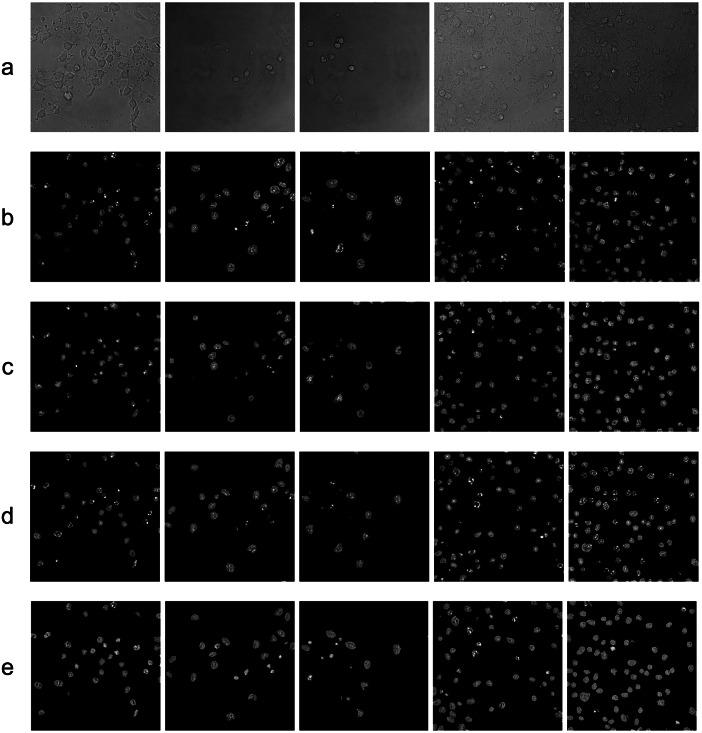
Translation results and comparison of self-attention cGAN (SAcGAN) model. (a) Middle slices of input bright-filed image stacks. (b) Ground-truth fluorescent images. (c) Results from pixel-to-pixel model. (d) Results from SAcGAN model (0011). (e) Results from XAcGAN model (0011).

For the SAcGAN model with one output channel, the architecture of the SAcGAN model could be seen as the XAcGAN model without the mask generation path and with the cross-attention modules replaced with self-attention modules. The structure for each sub-module and strategies for choosing the inserting position were the same. The performance of the SAcGAN model is listed in Table 3. From Table 3, we found the SAcGAN models did significantly better than the pixel-to-pixel model; among SAcGAN models, model 0011 provided the best performance in all three evaluation metrics. Figure 11 shows the fluorescent image translation result of the SAcGAN model 0011.

**Table 3.** Performance of self-attention cGAN (SAcGAN) model.

			Self-attention module inserted location			
Evaluation metrics	Pix2Pix	XAcGAN	0000	0001	0011	0111
L1_distance	0.0546	0.0256	0.0470	0.0470	0.0388	0.0492
PSNR	20.5644	23.6517	21.6721	21.4682	22.8019	21.3259
SSIM	0.8494	0.9310	0.8741	0.8726	0.8897	0.8673

Abbreviations: PSNR, peak signal-to-noise ratio; SSIM, structural similarity index measure.

Statistically speaking, compared to the SAcGAN model, the result of the XAcGAN model which contained mask generation was not overwhelmingly better through the evaluation metrics. However, the function of predicting the state of cells or nuclei was irreplaceable and this is the main advantage of our model. To evaluate the accuracy of nuclear detection and classification, the state of nuclei in the ground-truth fluorescent images was recorded and compared to detections in the translated images generated by the tested models. For all models, possible translation errors covered unexpected nuclei (false positive) and undetected nuclei (false negative). While for XAcGAN, it was also possible to evaluate the misclassification of nuclei (“healthy” or “apoptotic”), for the single output channel models (Pix2Pix and SAcGAN), this had to be done manually based on the evaluation of nuclear texture. In practice, nuclei from single output channel models were frequently difficult to classify in this manner due to ambiguous textures. As such, the reliability of counts for Pix2Pix and SAcGAN might not be highly accurate in Table 4 as it is affected by human factors but reflects the consequence of the no-mask-assistance model.

**Table 4.** Nuclei classification result.

	Figure no.		1	2	3	4	5	6	7	8
Ground truth	Healthy		91	19	19	9	37	78	26	34
	Apoptotic		6	17	5	7	29	6	4	13
XAcGAN (0011)	False positive		0	0	0	1	1	2	0	0
	False negative		4	0	1	1	3	1	1	7
	Mistakenly Classified	H to A^a^	4	2	1	0	0	1	0	
		A to H^b^	2	4	0	0	8	2	0	
Pix2Pix model	False positive		0	1	0	0	3	0	0	0
	False negative		1	0	3	1	0	1	5	13
	Unsure		12	11	3	2	29	5	3	4
SAcGAN (0011)	False positive		0	1	0	0	1	0	0	0
	False negative		1	0	1	0	0	3	0	1
	Unsure		23	10	2	2	20	13	6	12

a “H to A” represents healthy nuclei predicted to be apoptotic.

b “A to H” represents apoptotic nuclei predicted to be healthy.

Table 4 contains the detection and classification result of the XAcGAN 0011 model: the nuclei state prediction accuracy was 85.68%; the false positive error was 1.27%, the false negative error was 4.78%, and the state classification error for detected nuclei was 8.27%, which is comprised of a healthy-to-apoptotic error of 4.01% and apoptotic-to-healthy error of 4.26%. From Table 4, it is evident that the XAcGAN model performed significantly better. One reason was that our model was trained on the database with a reliable classification ground truth. This advantage came from the process of dataset pre-processing, in which we used the whole 13 slices of the fluorescent image stacks for mask generation. For the models without a predicted mask from the raw inputs, to perform state prediction, additional methods for classification were required. However, the classification was applied to predicted outputs, so it would be hard to guarantee the accuracy of the classification.

**Figure 12.**
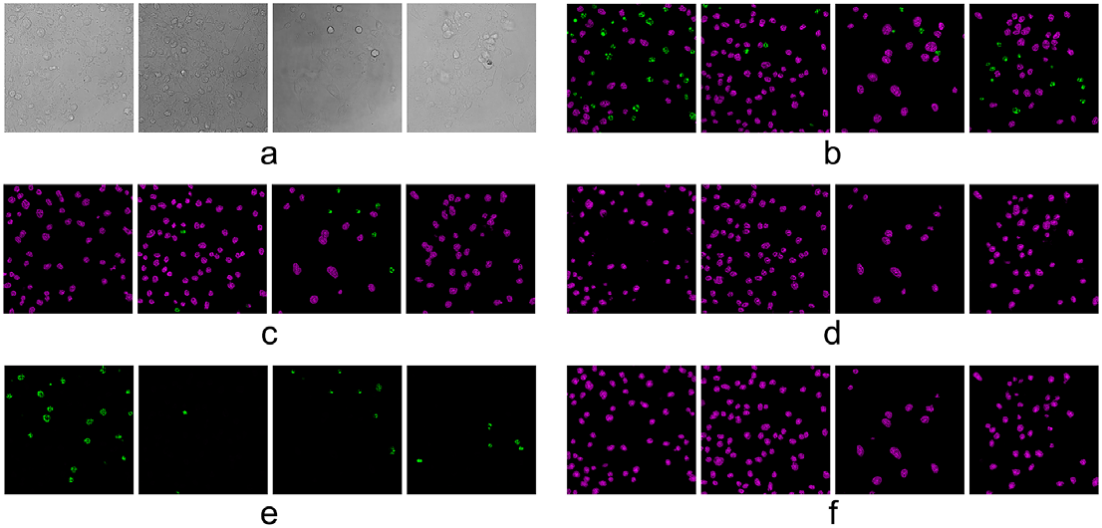
Translation results of self-attention cGAN model with two output channels (without mask generation path). (a,b) Middle slices of bright-field image stacks and corresponding ground-truth fluorescent images. (c–f) Results from the SAcGAN model which has no mask prediction path. (c) Model 0000 (no attention module applied). (d) Model 0001. (e) Model 0011. (f) Model 0111. Magenta nuclei indicate healthy nuclei and green nuclei indicate apoptotic nuclei.

We also tested the performance of SAcGAN with multiple output channels. In this test, each channel was aimed to present one state of nuclei, healthy or apoptotic. From Figure 12, we found that the model failed to produce fluorescent images. The results showed that the SAcGAN model tended to translate nuclei into one channel and ignore the generation in the other channel. For example, in Figure 12, only potential apoptotic nuclei were translated, and all the healthy nuclei were ignored. On the contrary, other models only recognized healthy nuclei. The reason the SAcGAN model was unable to process the classification could be that the loss function of the image generation task was too weak to lead the model to promote the classification result. To reduce the losses, the model tended to sacrifice one channel’s output and get stuck in a local minimum. The mask generation path assisted the model in skipping the local minimum and guided the image translation path on which channel to translate the nuclei. This task was used as a comparison of the multi-output-channel model with and without a mask generation path, and to explore the necessity of information connection between the mask generation and image generation.

## 7. Conclusion

Our XAcGAN model achieved excellent performance in bright-field to fluorescent image translation tasks and provided promising health state prediction simultaneously. cGANs are a powerful tool in supervised image translation. The shortage of datasets didn’t affect the performance of our network. Attention-based modules applied in our model improved the performance of the image translation. Our model does not need multiple spatial extents as input, the attention modules encouraged the model to learn the long-distance spatial dependencies. Meanwhile, the cross-attention modules combined dependencies from both generation paths. The segmentation path was a crucial auxiliary for multi-state fluorescent image translation, as it guided the network on which channels the items to be generated should be assigned to. In addition, the prediction result can be used as a supplementary for healthy state evaluation.

For the biological research of monitoring the health state of cells and nuclei over time, the model is an alternative to the time and labor-consuming process of fluorescent labeling. It will reduce the work for microscopy experiments where only bright-field imaging is adequate for nuclei observation. This would be the most significant contribution of our research to cellular biology studies.

## Data Availability

Code for training and for using the pre-trained model for translation is available on GitHub at https://github.com/SpikeRXWong/fluorescent_image_translation.git. All the images used in this work can be accessed at https://doi.org/10.5523/bris.2w8trsx55b0k22qez7uycg9sf4.
